# Neuroprotective Drug for Nerve Trauma Revealed Using Artificial Intelligence

**DOI:** 10.1038/s41598-018-19767-3

**Published:** 2018-01-30

**Authors:** David Romeo-Guitart, Joaquim Forés, Mireia Herrando-Grabulosa, Raquel Valls, Tatiana Leiva-Rodríguez, Elena Galea, Francisco González-Pérez, Xavier Navarro, Valerie Petegnief, Assumpció Bosch, Mireia Coma, José Manuel Mas, Caty Casas

**Affiliations:** 1 Institut de Neurociències (INc) and Department of Cell Biology, Physiology and Immunology, Universitat Autònoma de Barcelona (UAB) & Centro de Investigación Biomédica en Red sobre Enfermedades Neurodegenerativas (CIBERNED), Bellaterra, Barcelona, Spain; 2 Hand and Peripheral Nerve Unit, Hospital Clínic i Provincial, Universitat de Barcelona, Barcelona, Spain; 3 Anaxomics Biotech, S.L, C/Balmes 89, 4º 2º, 08008, Barcelona, Spain; 4 Institut de Neurociències (INc), Department of Biochemistry and Molecular Biology, UAB, Bellaterra, Barcelona, Spain; 5 Department of Brain Ischemia and Neurodegeneration, Institute for Biomedical Research of Barcelona (IIBB), Spanish Research Council (CSIC), Institut d’Investigacions Biomèdiques August Pi Sunyer (IDIBAPS), Barcelona, Spain; 6 INc and Department of Biochemistry and Molecular Biology, UAB and CIBERNED, Bellaterra, Barcelona, Spain; 7 ICREA, Passeig Lluis Companys 23, 08010 Barcelona, Spain

## Abstract

Here we used a systems biology approach and artificial intelligence to identify a neuroprotective agent for the treatment of peripheral nerve root avulsion. Based on accumulated knowledge of the neurodegenerative and neuroprotective processes that occur in motoneurons after root avulsion, we built up protein networks and converted them into mathematical models. Unbiased proteomic data from our preclinical models were used for machine learning algorithms and for restrictions to be imposed on mathematical solutions. Solutions allowed us to identify combinations of repurposed drugs as potential neuroprotective agents and we validated them in our preclinical models. The best one, NeuroHeal, neuroprotected motoneurons, exerted anti-inflammatory properties and promoted functional locomotor recovery. NeuroHeal endorsed the activation of Sirtuin 1, which was essential for its neuroprotective effect. These results support the value of network-centric approaches for drug discovery and demonstrate the efficacy of NeuroHeal as adjuvant treatment with surgical repair for nervous system trauma.

## Introduction

Common diseases of the central nervous system (CNS), including psychiatric disorders and neurodegeneration, are caused by multiple molecular abnormalities as opposed to individual defects. Likewise, recovery from CNS trauma requires multiple strategies encompassing neuroprotection and repair and regeneration of CNS cells. It follows that effective therapies must target multiple pathways rather than single proteins. Systems biology is an indispensable analytical tool in drug discovery for complex diseases. First, it allows for a necessary shift from a gene-centric to a network-centric view that takes protein targets back to their physiological context, such that a systemic perspective of the environment is gained without losing molecular details. Second, it facilitates the generation of multi-component therapies by repurposing existing drugs with well-established safety, bioavailability, and pharmacology profiles.

Here we report the discovery of a neuroprotective and pro-regenerative drug combination with the therapeutic performance mapping system (TPMS), a platform for drug discovery based on systems biology and artificial intelligence (www.Anaxomics.com). TPMS facilitates the screening of drugs for their capacity to shift the profile of topological molecular maps from pathological to beneficial^1^. We used TPMS to identify known drugs likely to be beneficial in treatment of peripheral nerve lesions caused by trauma, tumors, or autoimmune reactions. The economic cost of treating injured patients is considerable due to both direct and indirect expenses, as the injuries tend to cause functional inability in previously productive people^2^. Nerve root avulsion (RA) leads to the most severe degree of nerve injury because nerves are completely separated from the spinal cord and sensory ganglia, thus causing loss of motor, sensory, and autonomic functions in the affected extremities^3^. RA often leads to deafferentation pain that may develop into central sensitization and severe neuropathic pain that is refractory to pharmacotherapy^4^. Detached nerves may be reimplanted but successful repair is time-dependent due to the existence of massive retrograde degeneration of motoneurons (MNs)^5^. Thus, effective therapeutic agents should target multiple mechanisms in order to maintain MN viability, promote regenerative capabilities, and minimize glial reactivity.

In order to discover such a multifunctional therapy, we built molecular maps using TPMS and quantitative and unbiased proteomic data obtained from a pre-clinical rat model of RA that leads to retrograde degeneration of MNs and from a rat model of distal axotomy (DA) and suture that leads to MN survival and nerve regeneration^6^. We screened these maps for neuroprotective combinations of FDA-approved drugs, and we validated the selected combinations in the RA model. We found that the combination of acamprosate (ACA) plus ribavirin (RIB), which we called NeuroHeal, promotes neuroprotection, nerve regeneration, and functional recovery and, unlike existing drugs, is not pro-nociceptive. The mechanism of action of NeuroHeal involves sirtuin 1 (SIRT1), an actively pursued therapeutic target. NeuroHeal thus warrants further evaluation for early treatment after peripheral nerve and RA injuries.

## Results

### Identification of putative neuroprotective drug combinations

**Figure 1:**
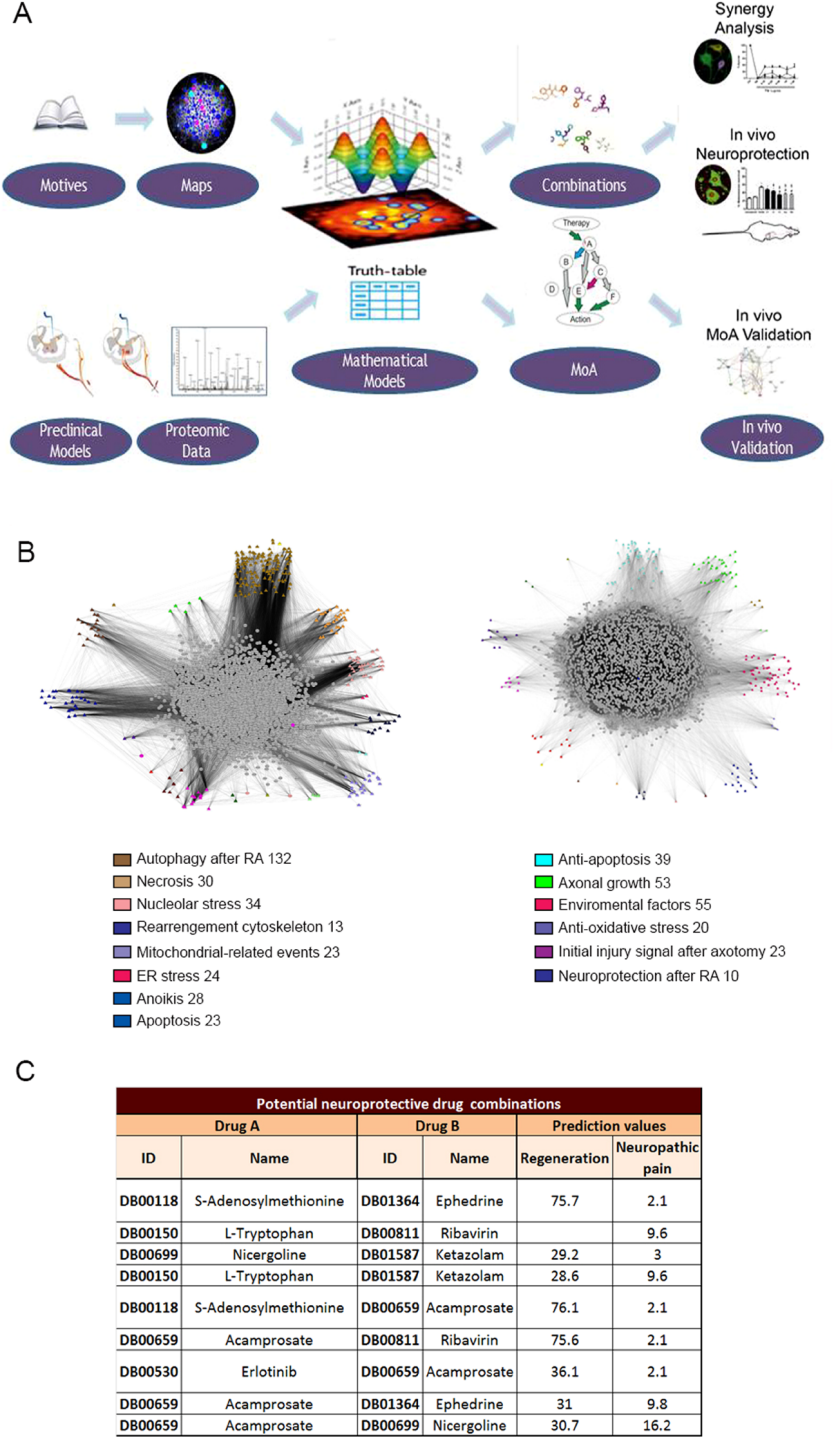
Experimental design. (**A**) The starting material was a manually curated list of key proteins clustered in motives that allowed construction of condition-specific networks for neurodegeneration after RA and for neuroprotection after DA. Using TPMS, network static maps were converted into topological maps associated with mathematical equations. The available data from unbiased proteomic analysis generated from RA and DA models (Casas *et al*., 2015) was used to build a set of restrictions collated into a truth table with which all models generated had to comply. Drug screening *in silico* was used to perturb the neurodegeneration-associated mathematical model and drug combinations that approximated the model to the neuroprotective state were identified. The algorithms used also allowed specification of key proteins involved in the mode of action (MoA) of each drug combination. Finally, we validated new combinations for its neuroprotective effect and putative MoA *in vivo* and *in vitro*. (**B**) Snapshots of the full protein networks associated with the neurodegenerative condition after RA (left, 3,836 nodes, average links per node 13.4) and with the neuroprotective condition after DA (right, 3,296 nodes, average links per node 13.9) visualized through the Cytoscape software platform^62^. Seed proteins for different motives are labelled by colour as indicated. Some seeds belong to more than one motive. (**C**) List of potential neuroprotective drug combinations identified using the *in silico* screen.

To identify potential neuroprotective drug combinations, we applied machine-learning tools as depicted in Fig. 1A. To generate our systems biology-based networks, the starting material was a manually curated list composed of proteins clustered in motives likely to be involved in either “neuroprotection” or “neurodegeneration” and obtained from a perusal of the literature in PubMed (Supplementary Tables 1 and 2). The initial list was expanded to generate network maps that included 3,296 proteins for regeneration and 3,836 proteins for degeneration with an overlap of 2,232 proteins. Snapshot of the maps are shown in Fig. 1B. The maps were converted into mathematical models incorporating all biological knowledge available including drug targets and clinical trials results^1,7–9^. Drug repositioning solutions were acquired by perturbing the models with stimulus (drugs) and approximating the best solution to the neuroprotection model. We incorporated our experimental proteomic data from the RA and DA models previously published^6^ and categorized as degeneration and neuroprotection conditions, respectively, for machine training to generate physiological responses facing any perturbation. These data resulted in a set of restrictions that all mathematical solutions should accomplish. In addition, the drugs screened were required to: i) have an outstanding safety profile, ii) not cause neuropathic pain, iii) have no known effects on CNS/PNS regeneration, and iv) be able to cross the brain-blood barrier. A total of 5,440 drugs that generated approximately 15 million binary combinations were screened. The threshold for drug candidates is calculated based on the cross-validation of the approved indications for each drug. We obtained 33 combinations that accomplished predicted values for peripheral nerve regeneration of 23%, for neuropathic pain of 17% or lower and more than 20% of synergism. Among those, several combinations contained at least one compound that had reported effects over regenerative properties. Hence, we further selected those combinations with standout safety profile and priority art. From the scored resulting binary combinations (Fig. 1C) we selected the top 3, having more than 75% of potential regenerative capabilities and less than 2.5% relation with neuropathic pain formation, for further experimental validation: ACA plus RIB (Combination C1); ACA plus ephedrine (EPHE) (C2), and S-adenosylmethionine (SAM) plus EPHE (C3).

### *In vitro* and *in vivo* validation of drug combinations

**Figure 2:**
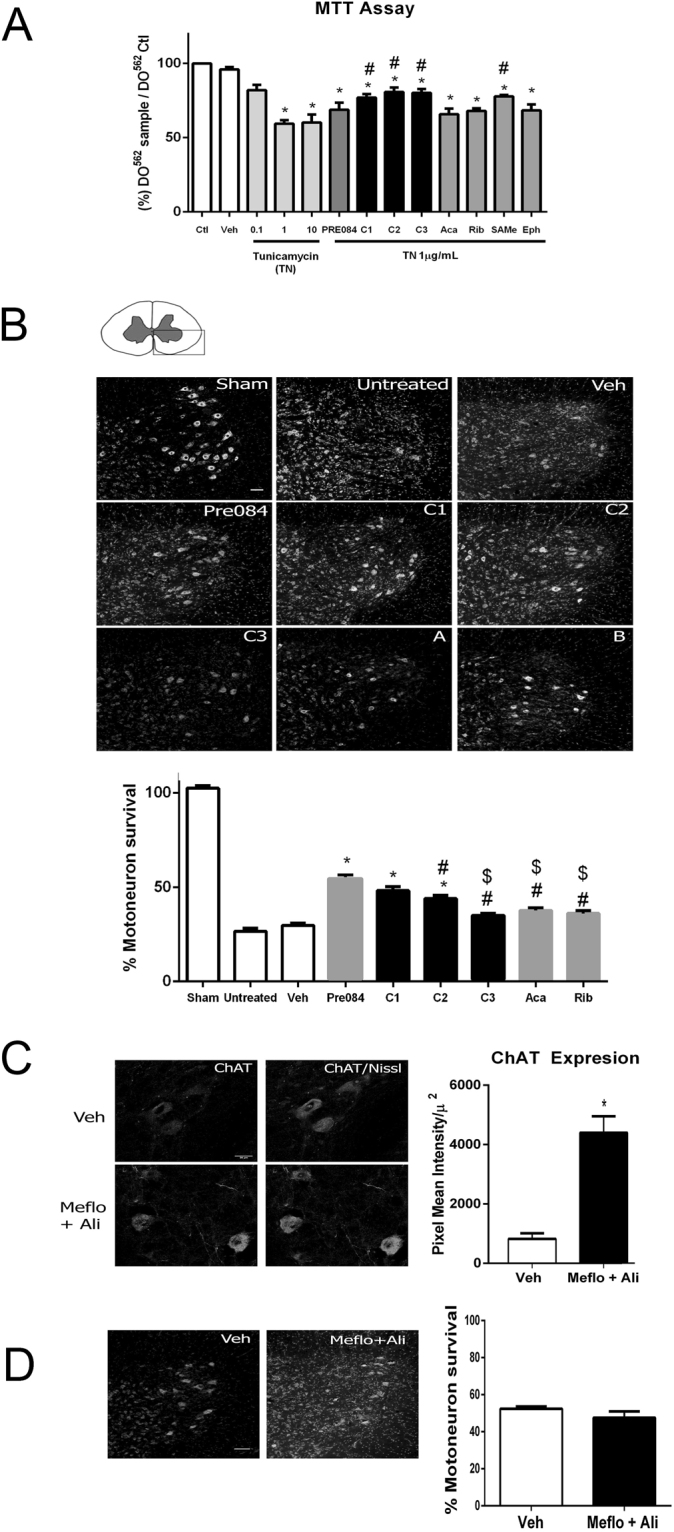
Neuroprotection by drug combinations identified *in silico*. (**A**) Bar graph showing the percentage of cell survival ± SEM after treatment with different doses of TN, which causes ER stress, to establish the optimal concentration to be used *in vitro* (fixed at 1 µg/ml TN). Neurotoxicity was evaluated with an MTT assay on differentiated NSC-34 MN-like cells in the absence of treatment (control, ctrl) or vehicle (veh) or presence of a single drug (Pre084, ACA, Rib, SAM, or EPHE or drug combinations (C1-C3) analysed 24 h after adding treatments (n = 3–8, *p < 0.05 vs. vehicle, ^#^p < 0.05 vs. 1 µg/ml TN). (**B**) *Top*, representative microphotographs of spinal cord ventral horns at L4-L5 from sham-operated control or the ipsilateral side of RA animals stained with fluorescent Nissl. Animals were intrathecally treated using programmable infusion pumps with either vehicle (artificial cerebrospinal fluid), PRE084 (positive control), single drugs, or combination of drugs: C1 = ACA (drug A) + RIB (drug B); C2 = EPHE + ACA; and C3 = SAM + EPHE. Scale bar = 100 µm. *Bottom*, bar graph of the average relative number of surviving motoneurons ± SEM on the ipsilateral side with respect to the contralateral side after 21 days post injury (dpi; n = 3 for Sham, PRE084, C3; n = 6 for injured; n = 4 for other groups, ANOVA, post hoc Bonferroni ^*^p < 0.05 vs. vehicle, ^#^p < 0.05 vs. PRE084, ^$^p < 0.05 vs. C1). (**C**) Microphotographs of ChAT immunohistochemistry in the ventral horn MNs from RA animals treated with vehicle (veh) or with a non-related drug combination of Mef and Ali at 14 dpi. Ali increases expression of ChAT. Bar graph of immunoreactivity intensity per area within MNs (Nissl-positive). (n = 3). (**D**) Representative microphotographs of MNs of the ipsilateral ventral horn stained by Nissl. Bar graph showing the average number of MNs ± SEM on the ipsilateral with respect to the contralateral side of spinal cord from animals treated with either vehicle or the combination of Mef and Ali at 21 dpi. (n = 3).

To validate the neuroprotective effect of the drug combinations and the synergy between their components we used an *in vitro* model of endoplasmic reticulum (ER) stress, since this is a hallmark of the RA neurodegenerative process^10^. We treated differentiated NSC-34 MN-like cells with tunicamycin (TN, an ER stressor), vehicle, individual drugs, or one of the drug combinations (C1-C3) and assessed viability with the MTT assay. Drug combinations, but not single drugs with the exception of SAM, protected the cells against ER stress (Fig. 2A).

We next performed RA in animals that received treatment with vehicle, single drugs, or drug combinations using subcutaneous implanted programmable pumps for continuous intrathecal infusion during 20 days post-injury. We used Pre084, a selective agonist of receptor σ1^11^, as a positive control of neuroprotection; Pre084 has no clinical value as it is pro-nociceptive^12,13^. Among all combinations, only C1(ACA + RIB) yielded a similar rate of MN survival as Pre084. Treatment with single C1 components ACA or RIB alone did not promote neuroprotection (Fig. 2B). Next, in order to determine the specificity of neuroprotection, we treated RA animals with a non-relevant combination composed of meflokine (MEF) and alitretionion (ALI), which we previously discovered using a similar systems biology approach for amyotrophic lateral sclerosis^1^. The drug combination reached its target after a continuously pumped infusion because there was upregulation of choline acetyl transferase (ChAT) expression within the MNs of treated RA animals (Fig. 2C) as previously demonstrated^1^. However, the combination was not neuroprotective (Fig. 2D), suggesting that the results obtained with TPMS were highly pathology-specific.

**Figure 3:**
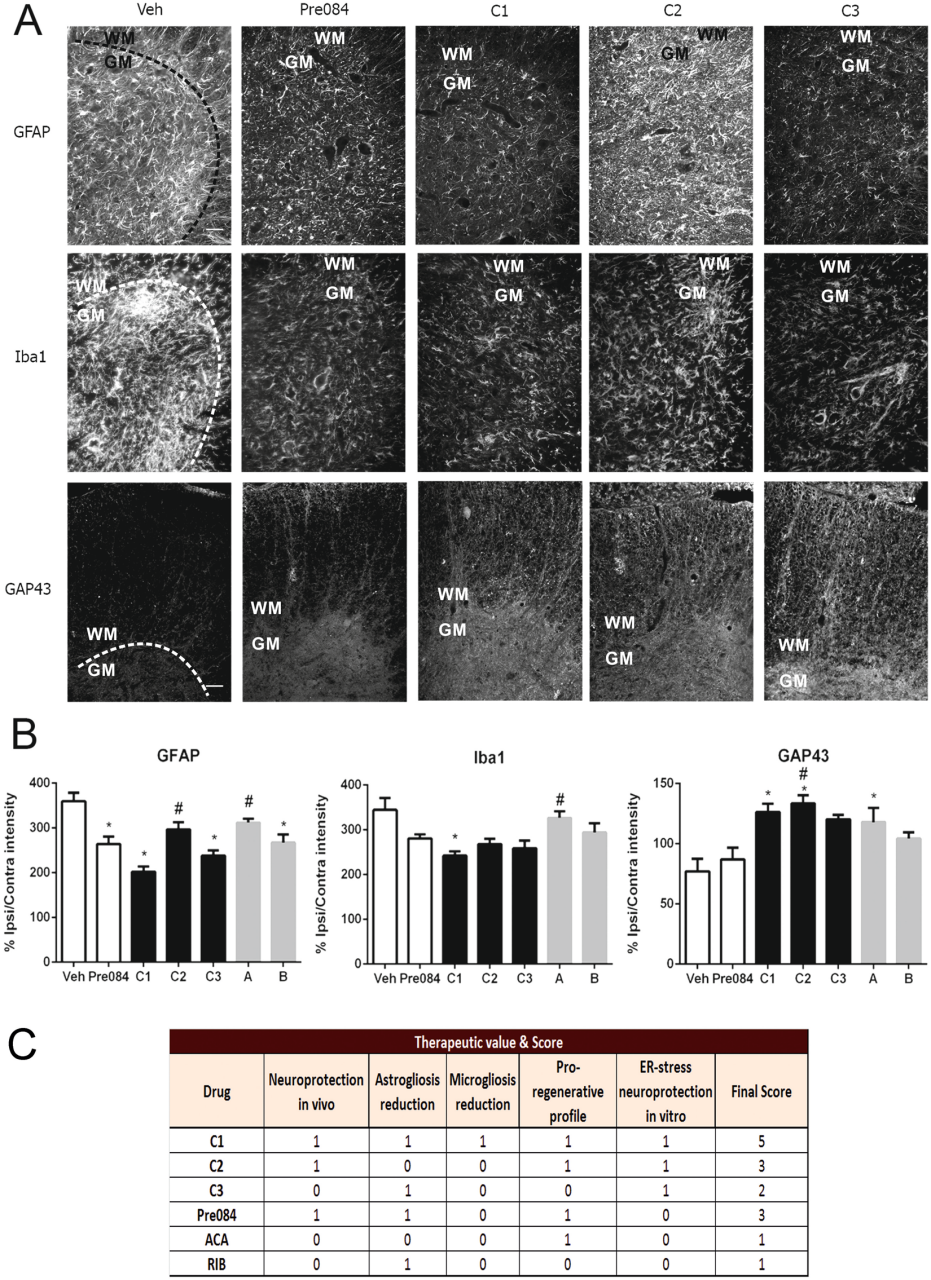
All drug combinations reduce microgliosis and astrogliosis and promote neuronal regeneration. (**A**) Representative fluorescence microphotographs at low magnification of the ipsilateral ventral horns of the spinal cord from RA injured animals treated with vehicle (Veh), Pre084, or drug combinations (**C**). Top and middle panels, staining for astrocytes (GFAP) and (middle panel) microglia (Iba1), respectively, in grey matter (GM)—delimited with dashed lines. Bottom panels, GAP43-positive neurites at the white matter (WM) of the ipsilateral ventral horns. Scale bar = 100 μm. (**B**) Bar graph of average immunoreactivity intensities in GM for GFAP and Iba1 and in WM for GAP43 (*p < 0.05 vs. Veh, ^#^p < 0.05 vs. C1). (n = 4). (**C**) Table summarizing dichotomy scores for MN survival, gliosis, and pro-regenerative effects *in vivo* and neuroprotective effects *in vitro* (1 is beneficial effect and 0 indicated no effect).

C1 and C3 treatments, in contrast to C2, reduced both microgliosis and astrogliosis after RA, as determined by immunohistochemical analysis of GFAP-positive astrocytes and Iba1-positive microglia (Fig 3A,B). Of drugs given individually only RIB slightly reduced astrogliosis (Fig. 3B). To examine the existence of a pro-regenerative profile within MNs, we analyzed the expression of GAP43, a protein associated with proliferation, in the motor axonal branches on the lateral-ventral side of the ipsilateral *versus* contralateral spinal cord sections. Most of the combinations analyzed and ACA alone, but not after Pre084 or RIB, increased GAP43 levels (Fig. 3B). We scored all drug combinations with regards to neuroprotection, reduction of inflammation, regeneration, and ER stress protection *in vitro* (Fig. 3C). C1(ACA + RIB) was the best in all readouts, and we named it NeuroHeal.

### Determination of drug synergy in NeuroHeal

**Figure 4:**
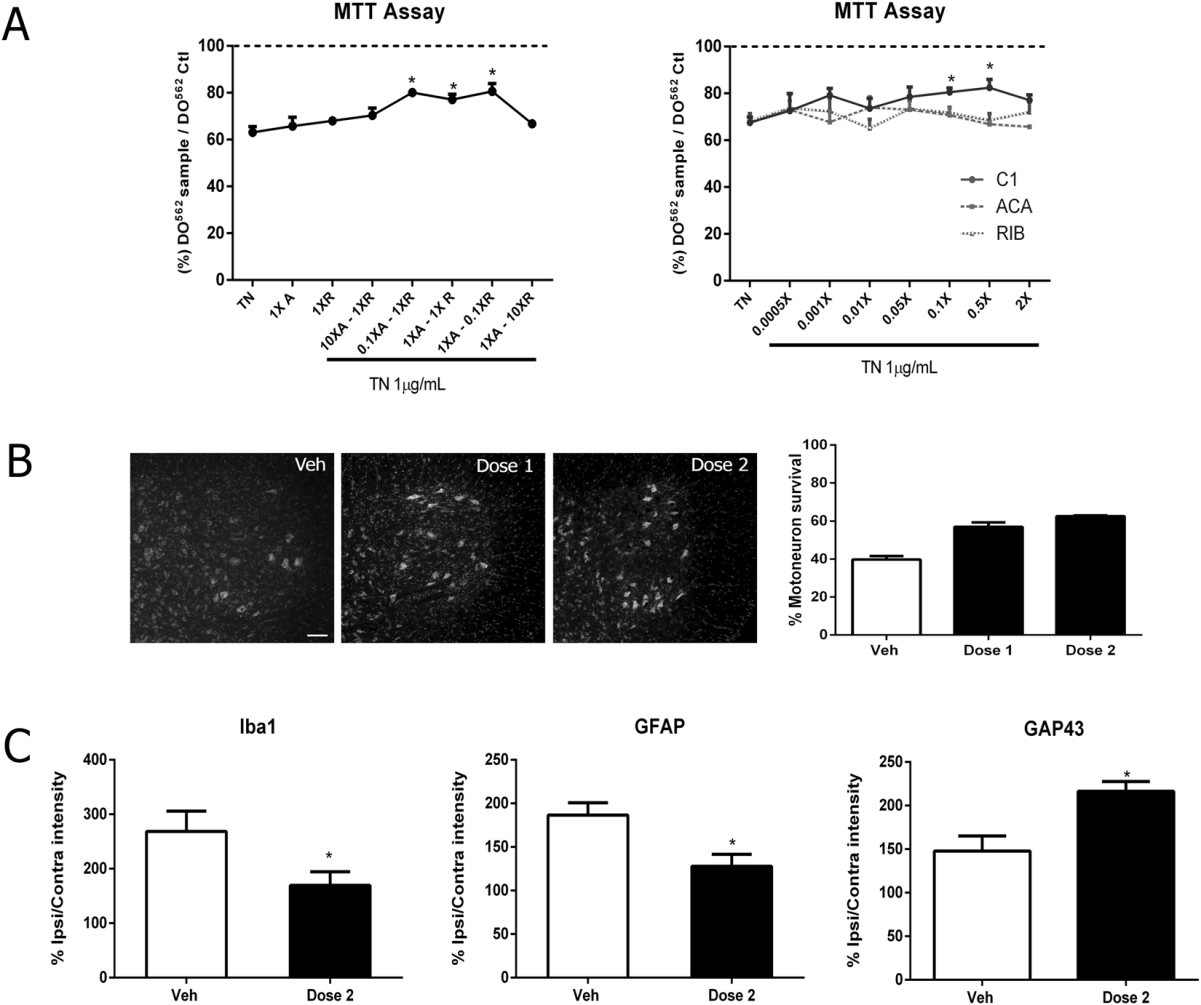
NeuroHeal has a supra-additive neuroprotective effect and is effective upon oral administration. (**A**) ***Left***, Bar graph of NSC-34 cell survival upon ER stress measured by MTT assay at different dose ratios of ACA (A; 1X = 0.22 mM) to RIB (R; 1X B = 4 µM). *Right*, Effect of range of doses within 0–2X with NeuroHeal or with single drugs at 1X (p < 0.05 with respect to TN alone). (**B**) Representative microphotographs of MNs stained by Nissl at the ipsilateral ventral horns of RA animals treated orally with either vehicle or dose 1 (0.22 mM ACA + 0.4 µM RIB) or dose 2 (0.06 mM ACA + 1 µM RIB), and bar graph of the percentage of surviving MN cells at the ipsilateral side with respect to the contralateral side. (n = 4). (**C**) Bar graph of average immunoreactivity intensity for GFAP, Iba1, and GAP43 in a fixed region of interest in the ipsilateral ventral horn in grey matter for GFAP and Iba1 staining or white matter for GAP43 (n = 4; *p < 0.05 vs. vehicle).

In order to determine the optimal dose combination we assayed ACA and RIB in different concentrations, changing by approximately 10-fold the amount of one drug with respect to the other. The fixed, 1X concentrations were 0.22 mM for ACA and 4 µM for RIB. We measured protection against cellular death caused by ER stress using the MTT assay. Neuroprotection was observed in a range of 0.1X to 1X for individual drugs, but not with higher doses (10X) (Fig. 4A, left). We next narrowed the range of concentrations from 0 to 2X. ACA or RIB treatment alone had no significant neuroprotective effect. In contrast, C1(ACA + RIB) afforded neuroprotection from 0.1X until less than 1X (Fig. 4A). Statistical analysis performed as previously described^14^ revealed a supra-additive effect when the drugs were combined to form NeuroHeal. Thus, for *in vivo* testing, we chose two doses with the best outcomes: 0.22 mM ACA plus 0.4 µM RIB and 0.06 mM ACA plus 1 µM RIB.

We administered these doses orally in RA injured animals because both drugs cross the blood-brain barrier in animals. The oral doses required to achieve effective of 0.22 mM ACA plus 0.4 µM RIB (dose 1) and 0.06 mM ACA plus 1 µM RIB (dose 2) in the CNS were estimated on the basis of previous pharmacokinetic studies of ACA and RIB in rats^15–18^. Both dose 1 and dose 2 caused a significant increase in MN survival as compared with vehicle-treated rats, although dose 2 was slightly more effective than dose 1 (Fig. 4B). We chose dose2 for further works and found that it reduced gliosis and promoted overexpression of GAP43 (Fig. 4C).

### NeuroHeal promotes functional recovery

**Figure 5:**
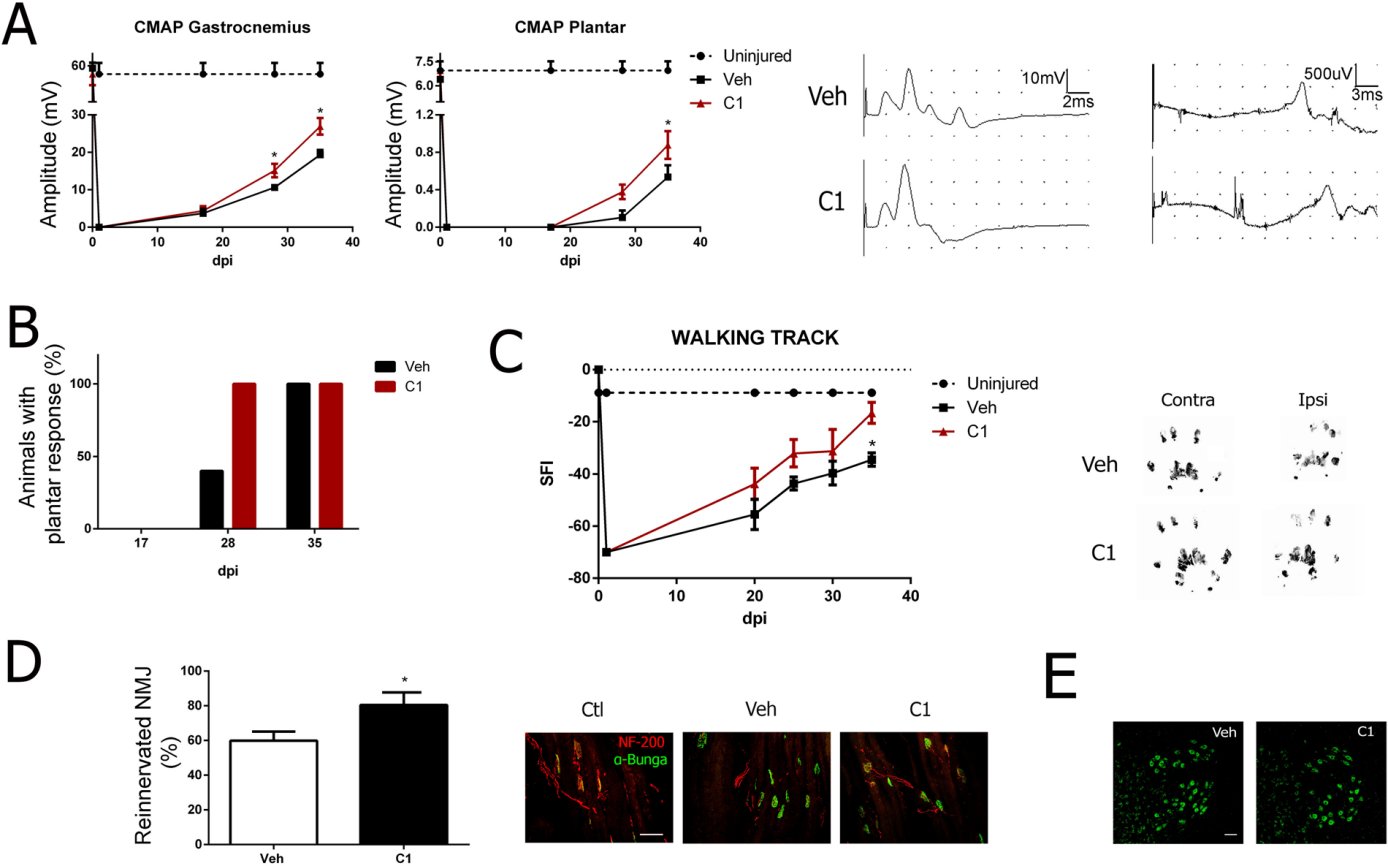
NeuroHeal accelerates nerve regeneration and improves muscle reinnervation and functional recovery after nerve crush injury. (**A**) *Left panels*, mean amplitudes of CMAP from ipsilateral gastrocnemius and plantar muscles after sciatic nerve crush of animals treated with vehicle (Veh) or NeuroHeal (C1; n = 5, ANOVA, post hoc Bonferroni*p < 0.05 vs. Veh). *Right panels*, representative recordings. (**B**) Histogram of the percentages of treated animals that presented electrophysiological evidence of reinnervation at the plantar muscle at different time-points. (**C**) *Left*, Plot of the sciatic functional index (SFI) obtained with walking track analysis of sciatic nerve in RA animals treated with either vehicle (Veh) or NeuroHeal (C1). *Right*, Representative footprints from ipsi- and contralateral paws at 35 days post injury (dpi). (**D**) *Left*, bar graph showing the percentage of reinnervated motor endplates at plantar muscle. *Right*, representative pictures of reinnervated neuromuscular junctions showing nerve fibers immunostained by NF200 (red) and end-plates labeled with bungarotoxin (green). (n = 4). (**E**) Microphotographs of spinal MNs stained with Nissl green at the ventral horn showing no signs of cell death due to nerve crush at 3 weeks post-injury.

The regenerative potential of NeuroHeal was confirmed in a model of crush injury of the sciatic nerve. The compound muscle action potentials (CMAP) evoked in response to sciatic nerve stimulation were recorded in gastronecmius and plantar muscles to assess functional recovery of denervated muscles. NeuroHeal led to a significant increase in CMAP amplitude in both muscles during the follow-up (Fig. 5A). After 28 days, there was reinnervation of the plantar muscle in all of the treated rats but in only 40% of the untreated (Fig. 5B), suggesting that NeuroHeal accelerated nerve regeneration. Furthermore, NeuroHeal improved the recovery of motor function, evaluated with the sciatic functional index (Fig. 5C), and increased the number of reinnervated neuromuscular junctions, assessed with co-localization of NF-200 with α-bungarotoxin (Fig. 5D). The numbers of MNs in rats after nerve crush were not significantly different in vehicle and NeuroHeal treated animals (Fig. 5E).

### The mechanism of action of NeuroHeal

**Figure 6:**
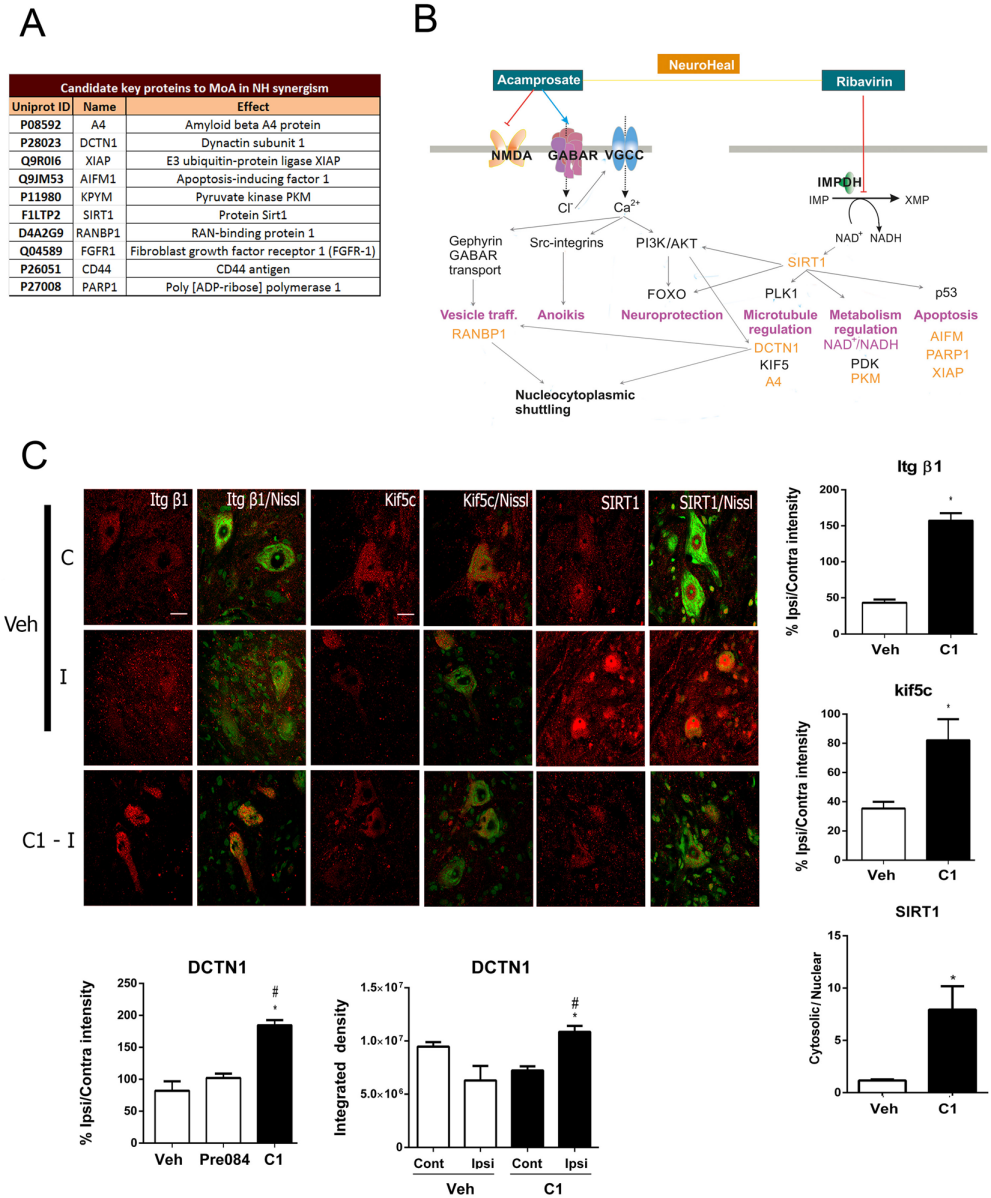
Molecular targets of NeuroHeal. (**A**) List of seed proteins predicted to be key synergic targets in the action of NeuroHeal. (**B**) Representation of putative NeuroHeal MoA from initial ACA and RIB targets to downstream possible effects to yield the synergic effects (pink) through its targets (orange). Representation is based on analysis using STRING and IntAct platforms and manual scrutiny of relevant literature. (**C**) *Left*, microphotographs of ipsilateral (I) and contralateral (**C**) ventral horns immunostained to reveal Itgb1, Kif5c, and SIRT1 in MNs (red) counterstained with green fluorescent Nissl (merged pictures) in animals treated with vehicle (Veh) or NeuroHeal (C1). Scale bar = 50 μm. *Right and Bottom*, bar graphs of the average ratios of immunofluorescence intensity (IF) between ipsi- and contralateral sides within an equivalent pre-determined region of interest (ROI) localized in the lateral grey matter for all conditions except for DCTN1, which was measured in the white matter (n = 4 animals, 5 MN/section, 3 sections, *p < 0.05). The bottom right histogram of DCTN1 analysis shows the quantification of total integrated intensity on each side to document that NeuroHeal only changes expression on the injured site (n = 4 animals, 5 MN/section, 3 sections, *p < 0.05).

TPMS analysis allowed the identification of putative proteins that mediate the synergistic mechanism of action (MoA) for NeuroHeal. The manually curated collection of molecular effectors (seeds) used in the model are listed in Fig. 6A. We used different platforms like STRING and IntAct as well as literature perusal to establish functional relationships among these proteins and the known targets of each drug composing NeuroHeal (Fig. 6B, Supplemental Figs 1 and 2). The known actions of ACA (DB00659) include the antagonism of the N-methyl-d-aspartate (NMDA) receptor and the metabotropic glutamate receptor 5 and the positive modulation of GABA receptor (A) (GABAR(A)). Hyperpolarization caused by Cl^−^ entry due to GABAR(A)) stimulation is normally proceeded by a depolarization caused by L-type voltage-gated calcium channel activation^19^. The consequent entry of Ca^2+^ may trigger different pathways, importantly PI3K activation^20^. PI3K activation may lead to the pro-survival AKT and FOXO pathway activation^21^ and also to an increase in cytoskeletal dynamics^22^. In particular, it may favor vesicle trafficking such as the gephyrin-mediated transport of GABA receptors to the surface membrane helped by dynactin, Kif5, and Hap1^23,24^ and nucleocytoplasmic shuttling mediated by Ran-binding proteins (RANBP)^25^. PI3K activation may also promote the activation of Src-integrin complex which in turn confers anti-anoikis properties^26,27^. In particular, the Src-integrin complex together with ranbp9 may favour endocytosis, which is anti-amyloidogenic^28^. Other ranbp proteins may be also activated, such as ranbp9, which is linked to active endocytosis and prevents the generation of amyloid peptide.

RIB (DB00811) inhibits inosine-5′-monophosphate dehydrogenase 1, an enzyme that catalyzes the conversion of inosine 5′-phosphate to xanthosine 5′-phosphate and acts as immunodulator^29^. Since this reaction consumes NAD^+^, its inhibition may lead to NAD^+^ accumulation. Hence, SIRT1, a nicotinamide adenine dinucleotide (NAD^+^)-dependent histone deacetylase and sensor of the NAD^+^/NADH balance^30^ may activate NAD^+^/NADH metabolism in a process involving PDK1 and PKM^31^. This leads to deacetylation of p53, which inhibits apoptosis^32^ and deacetylation and reinforced activation of AKT and FOXO^33,34^, which regulates microtubule dynamics resulting in neuroprotection in a process that involves PLK1 and Dynactin^35^. Indeed, PLK1 has been shown to reduce cell death mediated by amyloid peptide^36^.

In order to validate this putative MoA, we evaluated the effect of NeuroHeal on expression of some of these proteins such as the subunit b1 of integrin (ITGb1), kinesin family member Kif5c^6^, SIRT1, and the dynactin subunit DCTN1 in MNs at the ipsilateral site after 21 days post injury (dpi) in the RA model. We found that NeuroHeal increased the cytosolic expression of ITGb1, Kif5c, DCTN1, and SIRT1 in the ipsilateral horn with respect to the contralateral (Fig. 6C). Of note, the normalized levels of cytosolic SIRT1 in NeuroHeal treated rats was mostly due to the reduction in injury-induced nuclear expression of the protein. DCTN1 analysis showed that the expression of the target protein was modulated exclusively on the ipsilateral side, suggesting that the MoA of NeuroHeal may be specific to the damage context.

**Figure 7:**
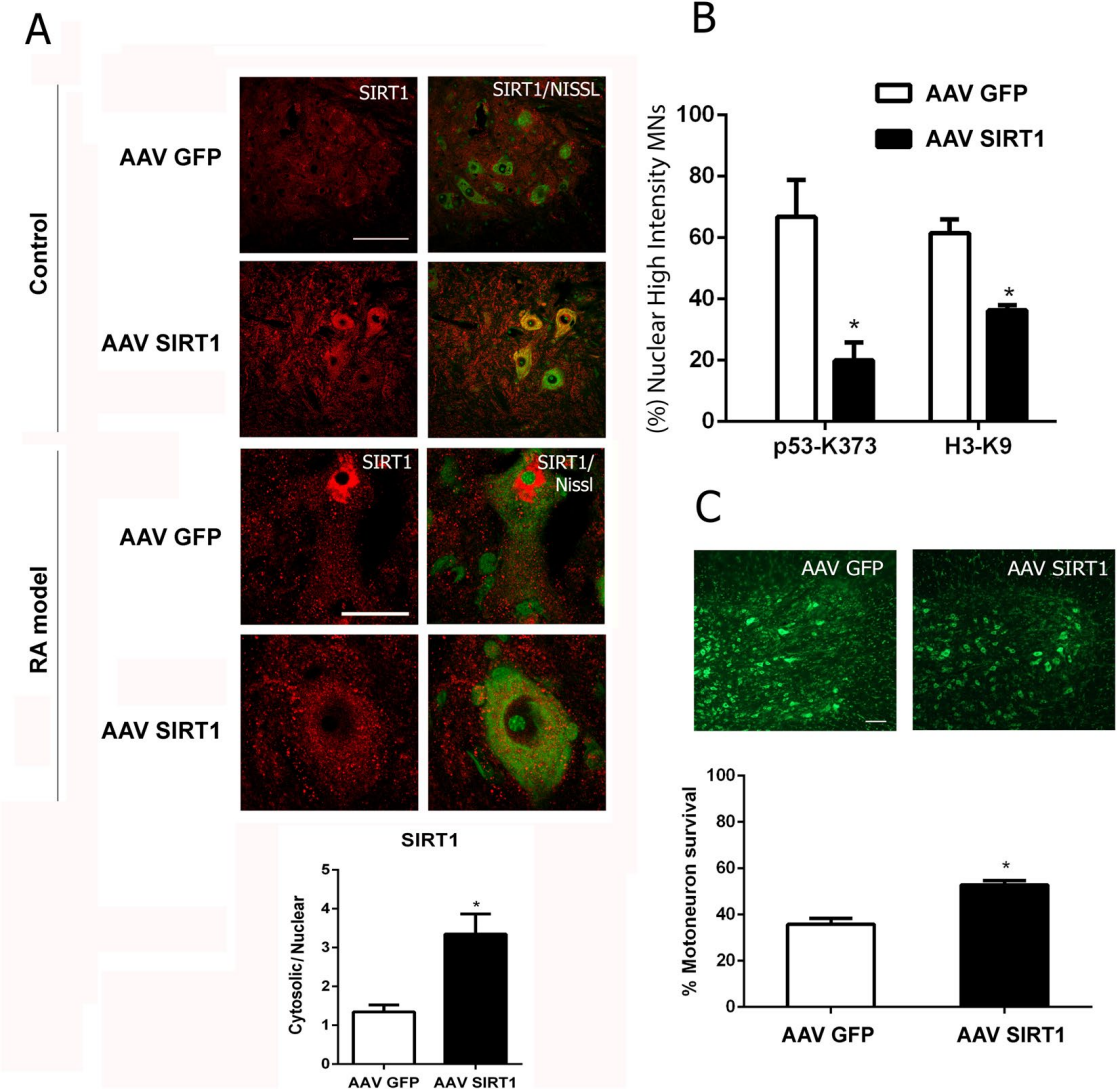
SIRT1 overexpression promotes MN survival after RA. (**A**) *Top*, representative microphotographs of SIRT1 immunolabelling (red) in infected MNs in control animals (*upper*) and RA injured animals (*lower*) treated with either AAVrh10-GFP or AAVrh10-SIRT1 and counterstained with green fluorescent Nissl. Scale bar = 100 µm (top); 25 µm (bottom). *Bottom*, histogram of cytosolic versus nuclear SIRT1 localization in the RA-injured MNs 21dpi after damage. (**B**) Histogram of the percentage of avulsed MNs with high nuclear immunofluorescence intensity for each acetylated form of either H3 (H3-K9) or p53 (p53-K373) at the ipsilateral side of RA animals infected with either vector (n = 4, *p < 0.05 vs. AAV-GFP). (**C**) Representative microphotographs of Nissl-labelled MNs (green) at the ipsilateral ventral horns of RA animals treated with either AAVrh10-GFP or AAVrh10-SIRT1and histogram of MN survival ± SEM expressed as % of MN on the contralateral side (contra) (n = 4, *p < 0.05 vs. AAVrh10-GFP).

Due to the importance of SIRT1 in many pathological conditions and in life span^37^, we investigated further whether SIRT1 was activated by NeuroHeal. First, we sought to determine whether viral overexpression of SIRT1 resulted in neuroprotection after RA since, due to NAD^+^ depletion promoted by SIRT1, SIRT1 does not always promote neuroprotection^30^. We cloned the *SIRT1* gene into a recombinant adeno-associated viral vector 10 (AAVrh10), which we previously had reported as being highly specific to MNs when intrathecally delivered to the spinal cord^38^. SIRT1 overexpression was localized in MNs, mainly in the cytoplasm (Fig. 7A). Within avulsed MNs, infection with AAVrh10-GFP did not change the nuclear ring-like pattern of SIRT1 expression induced by RA, but infection with AAVrh10-SIRT1 led to accumulation of SIRT1 predominantly in the cytoplasm of MNs, similar to NeuroHeal’s effect (Fig. 7A).

Second, we measured the deacetylase activity of SIRT1 by assessing the contents of histone-H3 acetylated at Lys9 (H3-K9) and of p53 acetylated at Lys373 (p53-K373) residues^39,40^. In RA animals treated with AAVrh10-SIRT1we observed: i) a decrease in both H3-K9 and p53-K373 acetylated forms, suggesting that SIRT1 overexpression caused an activity increase (Fig. 7B) and ii) a significant increase in MN survival (up to 52.90% ± 1.79) (Fig. 7C). Together these results suggest that overexpression and subsequent enhanced activation of SIRT1 is neuroprotective in severe peripheral nerve lesions.

**Figure 8:**
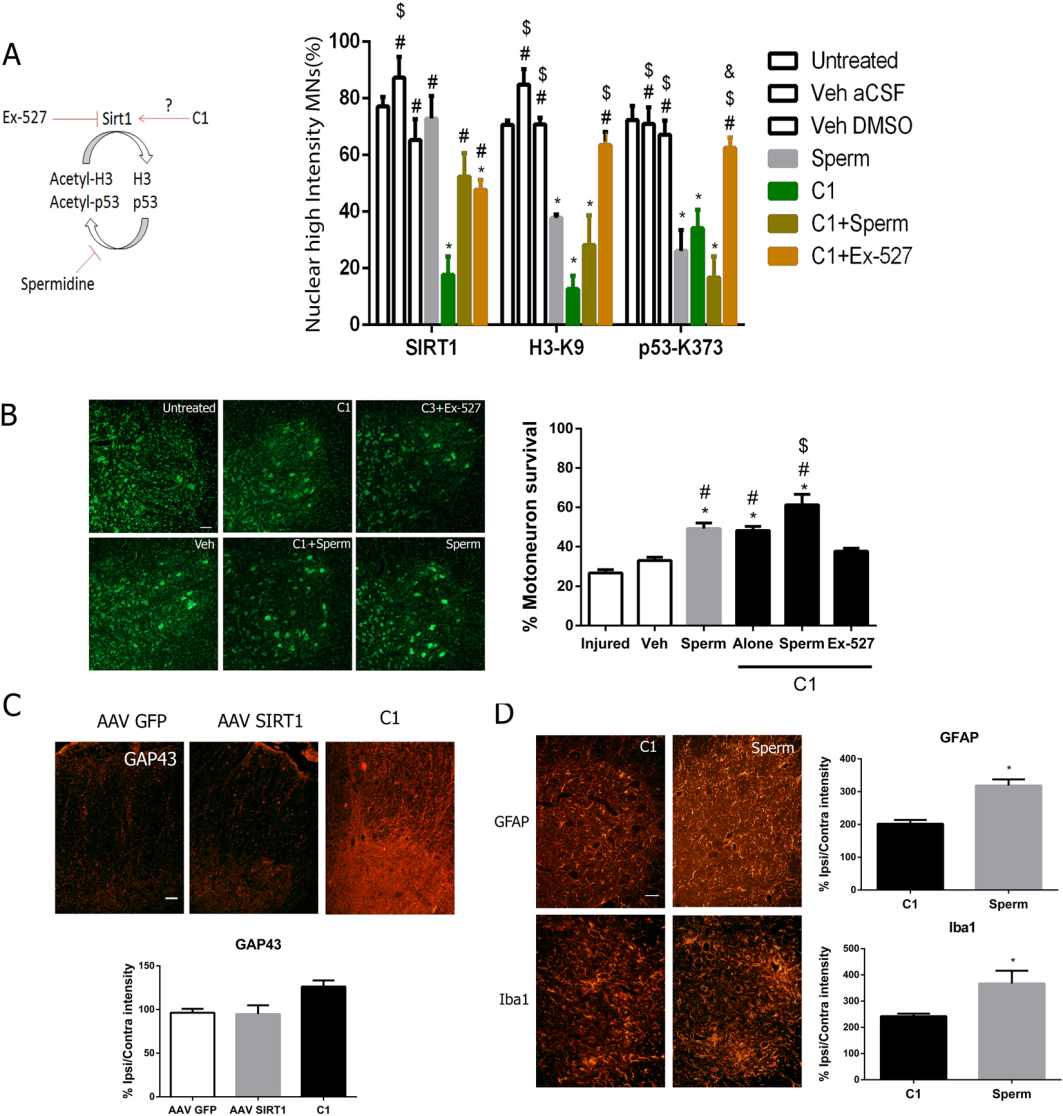
SIRT1 mediates the neuroprotective effect of NeuroHeal. (**A**) Diagram of the mechanisms of action of spermidine and Ex-527 and bar graph of the percentage of avulsed MNs with high nuclear immunofluorescence intensity for each marker on the ipsilateral side of RA animals treated with different drugs (n = 6 for untreated; n = 3 for Veh DMSO; n = 4 other groups, ANOVA, post hoc Bonferroni*p < 0.05 vs, ^#^p < 0.05 vs. C1, ^$^p < 0.05 vs. C1 + Sperm, ^&^p < 0.05 vs. Sperm). (**B**) Representative microphotographs of MNs on the ipsilateral sides and associated histogram of the average percentage of MN survival ± SEM in animals intrathecally treated with spermidine (Sperm) or Ex-527 with or without NeuroHeal (C1) (n = 4, ANOVA, post hoc Bonferroni *p < 0.05 vs. untreated, ^#^p < 0.05 vs. veh, ^$^p < 0.05 vs. C1 + Ex-527, *p < 0.05). Scale bar = 100 μm. (**C**) Microphotographs of GAP43 immunostaining at the ventral horns of the ipsilateral sides from animals treated with either AAVrh10-GFP, AAVrh10-SIRT1 or C1. Scale bar = 100 µm. Bar graph of the average immunoreactivity in a fixed region of interest of the white matter (n = 3–4, *p < 0.05 vs. AAVrh10-GFP). (**D**) Representative microphotographs of astrocyte (GFAP) or microglia (Iba1) staining at the ventral horns of the ipsilateral sides of RA animals treated with either NeuroHeal (C1) or spermidine (Sperm). Scale bar = 100 μm. Associated bar graphs of the average immunoreactivity in a fixed region of interest of the grey matter (*p < 0.05 vs. C1).

Third, we evaluated the effect of NeuroHeal in combination with either a specific inhibitor of SIRT1, Ex-527, which promotes the persistence of acetylated forms, or spermidine, an inhibitor of acetylases that causes the persistence of deacetylated forms^41^. Rats were treated with NeuroHeal and either Ex-527 or spermidine using the continuous intrathecal pump perfusion system for 20 days post RA injury. As readouts, we used nuclear SIRT1, H3-K9, and p53-K373 in injured MNs at the ipsilateral ventral horn. Maximal expression of all markers was observed in untreated and vehicle-treated mice. Spermidine did not alter SIRT1 distribution but, as expected, reduced H3-K9 and p53-K373 levels (Fig. 8A). NeuroHeal reduced nuclear levels of SIRT1 and acetylated forms of H3 and p53 and this effect was reversed by Ex-527 but not spermidine (Fig. 8A). Accordingly, spermidine, either alone or in combination with NeuroHeal, increased the survival rate of MN whereas Ex-527 abolished the neuroprotective effect exerted by the drug (Fig. 8B).

Finally, we compared the regenerative and anti-inflammatory capacities of NeuroHeal with those of virally transduced SIRT1 and spermidine. Although all treatments were neuroprotective, only NeuroHeal promoted GAP43 expression and reduced microgliosis and astrogliosis (Fig. 8C,D). These results demonstrate that deacetylation is important for the NeuroHeal-mediated neuroprotection after RA and that, synergistically acting through multiple targets, NeuroHeal performs better than single-target drugs.

## Discussion

Network medicine has become increasingly important for identifying novel disease mechanisms and predicting drug effects. This network-based approach enables elucidation of the underlying molecular mechanisms, mainly in terms of disease modules, disease phenotypes, and disease-disease associations^42–44^. A number of studies have investigated the disease modules associated with specific disease phenotypes such as asthma, diabetes, and cancer, for which a single disease module would mainly be detected^45,46^. That there should be a shift in the paradigm of drug discovery from a focus on single targets to the systems has been argued^47^, particularly in neuropathology^48^.

We reasoned that proteomic profiles from suitable preclinical models of MN neurodegeneration would provide unbiased data for use as input to machine learning and mathematical modeling to search for novel drug combinations. We took advantage of the existence of advanced molecular knowledge of the dissimilar reactions in MNs after either proximal or distal axotomy with opposite readouts (degeneration vs. survival, respectively). The rationale was that mimicking the endogenous mechanism that MNs engage in after distal axotomy should be neuroprotective. Computational tools available allowed us to screen for known drugs that *in silico* perturb the RA model to convert it into a DA model. We validated that some of the synergic drug combinations discovered *in silico* were neuroprotective in cell culture models and *in vivo*. The combination we call NeuroHeal had anti-inflammatory properties and induced pre-regenerative profiles in MNs. Furthermore, we identified the molecular downstream pathway that is modulated by NeuroHeal: SIRT1 is an important node in the network. Moreover, we validated that activation of SIRT1 mediated the neuroprotective action of NeuroHeal in a rat model of RA. To our knowledge this is the first work to demonstrate discovery of repurposed drug combinations using a network-centric approach with learning machine computational tools that moves forward from theory to practice and validates both the efficacy and mechanism of action using preclinical *in vivo* models. Previous partial studies have paved the way^49–51^.

Combined actions of ACA and RIB in NeuroHeal resulted in neuroprotection probably by exerting anti-apoptosis and anti-anoikis actions that we previously had shown to be key elements to activate the endogenous mechanisms of neuroprotection in axotomized MNs^6^. In the same report and in others^5^, the importance of cytoskeletal rearrangements for neuroprotection was also revealed; these rearrangements also appear to be facilitated by the NeuroHeal MoA. It would be interesting to validate the role of these and the others described processes in NeuroHeal MoA, similarly to what we have done for SIRT1.

NeuroHeal activated SIRT1 in damaged MNs after axonal disconnection. This is an interesting discovery because SIRT1 activators have long been sought due to the beneficial effects likely for several diseases^52–54^. Although SIRT1 is important for neurodegeneration^55^, it does not always lead to neuroprotection because it exhausts NAD^+^ resources^30^. The main advantage of NeuroHeal as compared to other recently discovered small-molecule sirtuin-activating compounds (e.g., STAC)^37^ is that its components have been proven to be safe for humans and cross the blood-brain barrier. Of note, NeuroHeal has effects beyond SIRT1 activation since it also promotes the expression of the pro-regenerative marker GAP43, while overexpression of SIRT1 does not. Finally, although we found that spermidine also affords neuroprotection of MNs after RA, its use in the clinic has been avoided as it is nociceptive^56^. Our work indicates that the role of epigenetic switches in neuroprotection deserves further investigation.

Unexpectedly, we found that NeuroHeal promoted nerve regeneration and functional recovery after nerve crush. This finding suggested that the proteomic data used from DA models may contain intrinsically pro-regenerative factors. It would be worth testing this hypothesis in more severe models to study CNS axon regeneration.

The properties of NeuroHeal make it a first-in-class therapeutic agent for nerve root disconnection or entrapments. The translation of NeuroHeal to the clinic will be facilitated because it is composed of already FDA-approved drugs. Knowledge about formulation is also advanced because we assessed the effect of different stoichiometry combinations of single drugs to achieve neuroprotective synergism.

We conclude that our network-centric discovery approach encompassing proteomic data relevant to disease and artificial intelligence is a powerful and promising methodology for the design of effective treatments based on drug repurposing. Repurposing speeds up the clinical translation of treatments for complex pathological conditions. Patent protection of NeuroHeal is currently in progress and funding is being raised in order to test the drug in a clinical trial.

## Materials and Methods

### TPMS technology

TPMS is a top-down systems biology approach with applications in drug repositioning ^57,19^. It is based on artificial intelligence and pattern recognition models that integrate all available biological, pharmacological, and medical knowledge to create mathematical models that simulate *in silico* the behaviour of human physiology. The process encompasses five steps: (i) A manually curated collection of molecular effectors (seeds) that characterize degeneration after RA and neuroprotection after DA, respectively, were created; (ii) condition-specific molecular maps were prepared from these seeds that incorporate all the available functional relationships; (iii) each static map was converted into mathematical models (topological maps) capable of reproducing existing knowledge and predicting new data; (iv) our own proteomic data from RA and DA models were used to feed machine learning and generate a set of restrictions that make up the truth table, and (v) mathematical models were solved to obtain multicomponent drug neuroprotective candidates for RA and a minimal description of its synergic MoA. For details see Supplementary materials.

### Subjects and surgical procedures

Sprague–Dawley female rats aged 12 weeks were kept under standard conditions of light and temperature and given food and water *ad libitum*. We performed surgical procedures under anaesthesia with a cocktail of ketamine/xylazine (0.1 mL/100 g weight) intraperitoneally (i.p.) as reported previously^5^. To perform extravertebral avulsion of the L4-L5 roots (the RA model) we made a midline skin incision and applied a moderate traction on the selected roots away from the intervertebral foramina, exposing the mixed spinal nerves that contained the motor and sensory roots and dorsal root ganglia. To carry out the sciatic nerve crush injury, we exposed the right sciatic nerve and crushed it in three different orientations using fine forceps (Dumont no. 5) for 30 seconds. The wound was sutured by planes and disinfected with povidone iodine, and the animals were allowed to recover in a warm environment. For intrathecal delivery of vehicle or drugs to the avulsed animals we used iPrecio programmable pumps (Data Science International, Italy), placed subcutaneously on the lumbar left side of the animal. The catheter connected to the pump was inserted into the magna cistern in the brain stem and fixed with surgical adhesive^58^. The pumps were programmed to release 30 µL from 18 to 20 hours after injury to reach the desired concentration in the CSF after a 1:5 dilution. Then, a continuous flow of 1 µL/h was released during 20 days from the day following RA until sacrifice to maintain the desired concentration in the CSF. All procedures involving animals were approved by the ethics committee (*Comissió d’Ètica i experimentació animal i Humana*) of the *Universitat Autònoma de Barcelona* and *Comité de Seguretat i Salut de la Generalitat de Catalunya*, and followed the European Council Directive 2010/63/EU.

### Electrophysiological and functional examination

See supplementary methods.

### Drugs

Pre084 (Tocris, Ellisville, MO, USA), mefloquine (Mef), alitetrinoin (Ali), S-adenosylmethionine (SAM), ephedrine (EPHE), acamprosate calcium (ACA), ribavirin (RIB; Norman), and Ex-527 (Sigma-Aldrich, Saint Louis, MO, USA) were diluted in artificial cerebrospinal fluid (aCSF: 124 mM NaCl, 3 mM KCl, 26 mM NaHCO_3_, 2 mM CaCl_2_∙2H_2_O, 1 mM MgSO_4_∙7H_2_O, 1.25 mM KH_2_PO_4_, and 10 mM D-glucose) used as a vehicle alone or with 0.01% DMSO in the case of comparative studies with Ex-527. The concentrations prepared in the pumps were 5 × the desired final concentration in animal CSF: 0.015 mM for MEF, 0.15 mM for ALI, 5 mM for ACA, 20 µM for RIB, 36.5 µM for EPHE, 187 µM for SAM, 50 µM for Pre084, and 7 mM for Ex-527. We added spermidine (Sigma-Aldrich) to the drinking water; it was freshly added at 30 mM concentration every 2–3 days for 21 days as described elsewhere^41^. For oral administration, ACA (Merck, Darmstadt, Germany) and RIB (Normon, Madrid, Spain) were dissolved in water at a final concentration of 2.2 mM and 1 mM, respectively for dose 2 (0.25 × group).

### Construction, purification, and infection with recombinant adeno-associated vectors

The *SIRT1* cDNA was cloned into *Nhe*I and *Xho*I sites between the ITRs of AAV2, under the regulation of *CMV* promoter and the woodchuck hepatitis virus responsive element (WPRE)^59^. AAV2/rh10 vector was generated as previously described^60^ by triple transfection of HEK 293-AAV cells (Stratagene, San Diego, CA, USA) with branched polyethylenimine (PEI; Sigma-Aldrich) with the plasmid containing the ITRs of AAV2, the AAV helper plasmid containing Rep2 and Cap for rh10 (kindly provided by JM Wilson, University of Pennsylvania, Philadelphia, PA, USA) and the pXX6 plasmid containing helper adenoviral genes^61^. Recombinant vectors were clarified after benzonase treatment (50 U/mL, Novagen) and polyethylene glycol (PEG 8000, Sigma-Aldrich) precipitation. Vectors were purified by iodixanol gradient by the Vector Production Unit at UAB (http://sct.uab.cat/upv), following standard operating procedures^61^. Viral genomes per ml (vg/ml) were quantified by picogreen (Invitrogen, Carlsbad, CA, USA).

Intrathecal administration of 4 × 10^−10^ viral genomes was performed at the lumbar region of isoflurane-anaesthetized animals using a 33-gauge needle and a Hamilton syringe. After lateral spine exposure by paravertebral muscle dissection, 10 µl of viral vectors were slowly injected into the CSF between vertebrae L3 and L4. Appropriate access to the intrathecal space was confirmed by animal tail flick. The needle was held in place at the injection site for one additional minute, after which muscle and skin were sutured.

### *In vitro* model

We used the NSC-34 motoneuron-like cell line cultured in Dulbecco’s modified Eagle’s medium high-glucose (DMEM, Biochrom, Berlin) supplemented with 10% fetal bovine serum and 1X penicillin/streptomycin solution (Sigma-Aldrich), on collagen-coated plates (Thermo-Fisher, Waltham, Massachusetts, USA) in a humidified incubator at 37 °C under 5% CO_2_. For ER stress we used added tunicamycin (0, 0.1, 1, or 10 µg/ml; Sigma-Aldrich) and MTT assay was performed as detailed in supplemenatry methods.

### Immunohistochemistry and image analysis

After deep anaesthesia with pentobarbital, we transcardially perfused the animals with a saline solution containing 10 U/ml heparin, followed by 4% paraformaldehyde in a 0.1 M phosphate buffer, pH 7.2 for tissue fixation at 21 dpi (n = 4 for each condition), and removed the L4 and L5 segments (5-mm total length) of the spinal cord, which were post-fixed in the same fixative for an extra 4 hours and cryopreserved in 30% sucrose overnight. Serial transverse sections (20-µm thick) were obtained on gelatinized slides using a cryotome (Leica, Heidelberg, Germany) and preserved them at −20 °C until use. For immunohistochemistry procedures see supplemental material. Sections to be compared were processed together on the same slide and on the same day. Images of the spinal cord samples from different treatments and controls were taken under the same exposure time, sensibility, and resolution for each marker analysed with the aid of a digital camera (Olympus DP50) attached to the microscope (Olympus BX51). We analysed signal intensity with the ImageJ software (National Institutes of Health; available at http://rsb.info.nih.gov/ij/). For GFAP and Iba1, microphotographs were taken at 40 × , and then we transformed them to grey scale and analysed immunoreactivity by measuring the integrated density of a region of interest (ROI) after defining a threshold for background correction^5^. The ROIs were selected on the grey matter at the ventral horn and had an area of 0.12 mm^2^ for GFAP and Iba1 and the same ROI size but in the white matter for GAP43 and DCTN-1. Measurements were performed from 8 spinal cord sections (separated 220 µm between pairs) of each animal.

Confocal microphotographs of nuclei of MNs were taken in identical conditions of exposure (Zeiss LSM 700; Zeiss, Jena, Germany). For SIRT1 and deacetylase activity substrate (p53K373 and H3k9) analysis, single-cell densitometry was performed by pre-defining the threshold for each section for background correction and measuring the total area of the encircled MN nucleus. Then the ratio of integrated density/area was used as an index to classify at least 15–20 MNs per section.

### Motor neuron counting

See supplementary methods

### Neuromuscular junction reinnervation analysis

We cut the plantar interossei muscles into serial transverse sections (40 µm thick) using a cryotome and preserved them at −20 °C until use. The slides were incubated with chicken anti-Neuro Filament 200 (NF-200; 1:1000, Millipore) as described above. After several washes Cy3-conjugated secondary antibody was added. Finally, we incubated slices with α-bungarotoxin labelling solution (Life Technologies) following the manufacturer’s protocol to reveal motor endplate machinery. Sequential microphotographs were taken covering all the plantar muscle at 20 × . Only motor end plates with NF-200 co-labelling were counted as reinnervated.

### Statistical analysis

All values are presented as means ± standard errors of the means (SEM). For statistical analysis, we analyzed data with GraphPad Prism 5 software (San Diego, CA, USA) using unpaired t-tests or one-way analysis of variances (ANOVA) followed by Bonferroni’s multiple comparison tests. We considered differences significant at p < 0.05.

## Electronic supplementary material

Supplementary information

Supplementary table 1

Supplementary table 2
