# Supplementary material for: Neuroprotective Drug for Nerve Trauma Revealed Using Artificial Intelligence
Source: Sci Rep. 2018 Jan 30;8:1879. doi: 10.1038/s41598-018-19767-3 (PMC5790005; doi:10.1038/s41598-018-19767-3)
Supplement: Supplementary file 1 — Supplementary information [file 41598_2018_19767_MOESM1_ESM.pdf]

Neuroprotective Drug for Nerve Trauma Revealed Using Artificial Intelligence

David Romeo-Guitart[1], Joaquim Forés[2], Mireia Herrando-Grabulosa[1], Raquel Valls[3], Tatiana Leiva-Rodríguez[1], Elena Galea[4], Francisco González-Pérez[1], Xavier Navarro[1], Valerie Petegnief[5], Assumpció Bosch[6], Mireia Coma[3], José Manuel Mas[3], Caty Casas[1*]

Affiliations

[1] *Institut de Neurociències* (INc) and Department of Cell Biology, Physiology and Immunology, *Universitat Autònoma de Barcelona* (UAB), & *Centro de Investigación Biomédica en Red sobre Enfermedades Neurodegenerativas* (CIBERNED), Bellaterra, Barcelona, Spain

[2] Hand and Peripheral Nerve Unit, Hospital Clínic i Provincial, *Universitat de Barcelona*, Barcelona, Spain

[3]Anaxomics Biotech, S.L, Barcelona, Spain

[4] *Institut de Neurociències* (INc), Biochemistry and Molecular Biology, UAB and *ICREA*, Passeig Lluís Companys 23, 08010, Barcelona, Spain

[5] Department of Brain Ischemia and Neurodegeneration, Institute for Biomedical Research of Barcelona (IIBB), Spanish Research Council (CSIC), *Institut d'Investigacions Biomèdiques August Pi Sunyer* (IDIBAPS), Barcelona, Spain

[6] INc and Department of Biochemistry and Molecular Biology, UAB and CIBERNED, Spain

*Correspondence should be addressed to: Caty Casas Louzao, *Unitat de Fisiologia Mèdica*, *Facultat de Medicina*, *Universitat Autònoma de Barcelona*, E-08193 Bellaterra, Barcelona, Spain. Tel: +34-935811324, Fax: +34-935812986, E-mail: Caty.Casas@uab.cat

Running title: Network-based drug discovery for CNS trauma

SUPPLEMENTARY INFORMATION

- **Supplementary Methods**
- **References**
- **Supplementary Tables and Headings**
- **Supplementary Figures and Legends**

SUPPLEMENTARY METHODS

TPMS technology (supplementary information)

Generation of molecular maps. We manual curated a list of proteins (seed proteins) relevant for the processes of neurodegeneration and neuroprotection (Supplemental Table 1 and 2). Manual curation was performed through an extensive and careful review of full-length articles in the PubMed database that included the strings defined in Figure 1B. The search was expanded using the "related articles" function and article reference list. The map generation and extension process was conducted through the incorporation of all known relationships of the seed proteins in the map based on the following sources: KEGG [1], REACTOME [2], BIOGRID [3], INTACT [4] [3], HDPR [5], MATRIXDB [6], MIPS [7], DIP [8], and MINT [9]. The final map included 12,000 proteins and 180,000 links connecting the proteins.

Generation and solving mathematical models. Models were generated through the use of ANN and pattern recognition (Sampling methods) techniques based on optimization of genetic algorithms [10,11]. The specific algorithm used for ANN was a multilayer perceptron (MLP) neural network classifier [12,13] with a backpropagation training method that typically consists of an input layer of nodes, an output layer, and one or more middle hidden layers of nodes in between [14]. The input layer is the raw data introduced. The learning methodology used consisted of an architecture of stratified ensembles of neural networks as a model, trained with a gradient descent algorithm to approximate values of a given truth table. MLP gradient descent training depends on randomization initialization. In order to generate each of the ensembles, 1000 MLPs were trained with the training subset. The best 100 were used as the ensemble. The model identified the relationships between drug targets and clinical elements of the network with a 98% of accuracy after applying cross-fold validation process. The truth table is a set of restrictions corresponding to the available biological knowledge about the constructed networks, together with the knowledge derived from DrugBank, GEO [15] and our own dictionary called Biological Effectors Database (BED) (http://www.anaxomics.com/our- technology/tpms/#tpms). The BED database contains the

molecular description and details of 212 clinical terms cited in DrugBank [16] summing more than 3500 proteins in 200 pathological conditions covering 98.5% of all clinical terms included [17]. We added to BED our own proteomic data from RA and DA models (Casas et al 2015). The models should be able to reproduce every single rule contained in the truth table so that the error of a model is calculated as the sum of all the rules with which the model did not comply. To validate our models, we used a previously reported neuroprotective drug (PRE084) as a positive control in our RA model [18].

Sampling methods were used to generate mathematical models with stratified ensembles that comply with the truth table. Each drug combination was considered an input signal that stimulates and changes the model. The integration of the input signals consisted of the sum of all input values that arrive at the node. This signal was submitted to a sigmoid function to produce a normalized output in the range [-1,1], this output-signal being the input-signal for the next node. The topology was initialised by random values for all the links in each of the 100 initial models. Each model is evaluated against the human functional description. In the present work the best model was that closest to the regenerative model. The worst models were discarded, and new models with different initialization values for the links were created. This iterative process was optimized by a stochastic optimization strategy exploring between 10^6 and 10^9 models as described previously [19]. The accuracy of the best 100 models improved with each new cycle of iteration. This process continued until the models did not improve with more cycles of iteration.

Models result in both "global" predicted mechanisms that account for the majority of the population, and "cluster" mechanisms of action, which are more accurate for population subgroups. The MoA was validated in a two-step process. First, we checked that each link was accurate (i.e., was already described in the literature). Second, we ensured that the MoA made sense overall, featuring pathways coherent within the living system. A normalized synergism score (SE) was obtained from the protein involved in the synergism between two drugs of the combination. SE, based on the ponderation of the number of solutions for a

particular node affected by both drugs, and the synergistic effect (rather than additive) of both drugs over the node, were determined with the maximum score according to this calculation being 0.5.

REFERENCES

1. Kanehisa, M. et al. From genomics to chemical genomics: new developments in KEGG. Nucleic Acids Res. 34, D354–D357 (2006).

2. Croft, D. et al. The Reactome pathway knowledgebase. Nucleic Acids Res. 42, D472-7 (2014).

3. Salwinski, L. et al. Recurated protein interaction datasets. Nature methods 6, 860–861 (2009).

4. Brooksbank, C. et al. The European Bioinformatics Institute's data resources. Nucleic Acids Research 31, 43–50 (2003).

5. Keshava Prasad, T. S. et al. Human Protein Reference Database--2009 update. Nucleic Acids Res. 37, D767–D772 (2009).

6. Chautard, E., Fatoux-Ardore, M., Ballut, L., Thierry-Mieg, N. & Ricard-Blum, S. MatrixDB, the extracellular matrix interaction database. Nucleic Acids Res. 39, D235-40 (2011).

7. Mewes, H. W. et al. MIPS: curated databases and comprehensive secondary data resources in 2010. Nucleic Acids Res. 39, D220-4 (2011).

8. Xenarios, I. et al. DIP: the database of interacting proteins. Nucleic Acids Res. 28, 289–91 (2000).

9. Licata, L. et al. MINT, the molecular interaction database: 2012 update. Nucleic Acids Res. 40, D857-61 (2012).

10. Goldberg, D. E. Genetic Algorithms in Search, Optimization, and Machine Learning. (Addison-Wesley).

11. Kirkpatrick, S., Gelatt, C. D. & Vecchi, M. P. Optimization by simulated annealing. Science 220, 671–80 (1983).

12. Cybenko, G. Approximation by superpositions of a sigmoidal function. Math. Control. Signals, Syst. 2, 303–314 (1989).

13. Rosenblatt, F. PRINCIPLES OF NEURODYNAMICS. PERCEPTRONS AND THE THEORY OF BRAIN MECHANISMS. (Spartan Books, 1961).

14. Arce-medina, E. & Paz-paredes, J. I. Artificial neural network modeling techniques applied to the hydrodesulfurization process. 49, 207–214 (2009).

15. Lopes, C. T. et al. Cytoscape Web: an interactive web-based network browser. Bioinformatics 26, 2347–2348 (2010).

16. Wishart, D. S. et al. DrugBank: a knowledgebase for drugs, drug actions and drug targets. Nucleic Acids Res. 36, D901-6 (2008).

17. Valls, R., Pujol, A. & Artigas, L. Anaxomics' methodologies: understanding the complexity of biological processes. white Pap. (2013).

18. Penas, C. et al. Sigma Receptor Agonist 2-(4-Morpholinethyl)1 Phenylcyclohexanecarboxylate (Pre084) Increases GDNF and BiP Expression and Promotes Neuroprotection after Root Avulsion Injury. J. Neurotrauma 28, 831–40 (2011).

19. Mitchell, M. An Introduction to Genetic Algorithms (Complex Adaptive Systems). The MIT Press (1998).

SUPPLEMENTARY FIGURES AND LEGENDS

Supplementary figure1

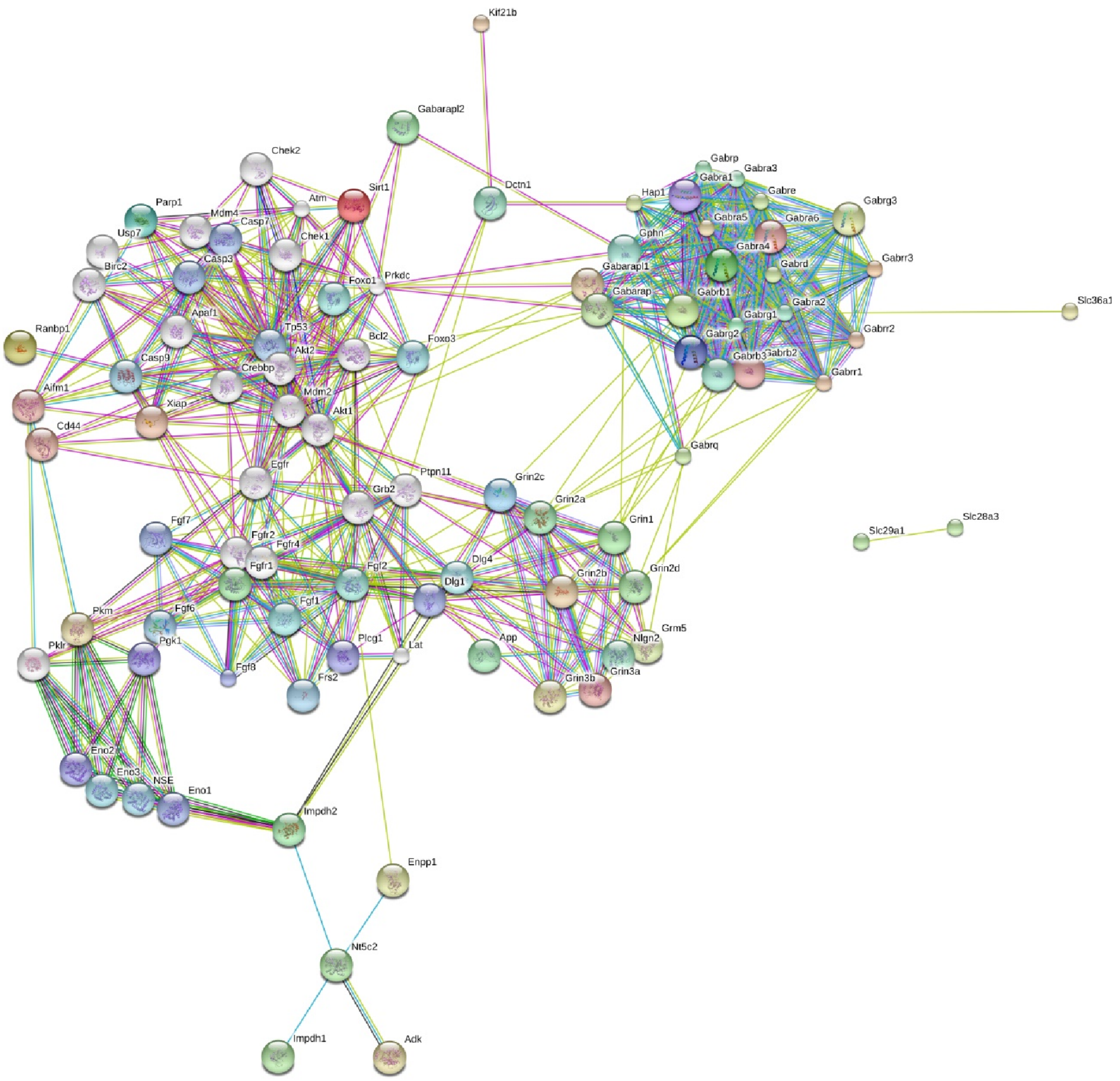

Supplemental Figure 1. Extended protein-protein interaction network resulting from STRING analysis with default parameters. Initial seeds used were NH synergic MoA-related proteins and known targets from ACA and RIB.

.

Supplemental Figure 2

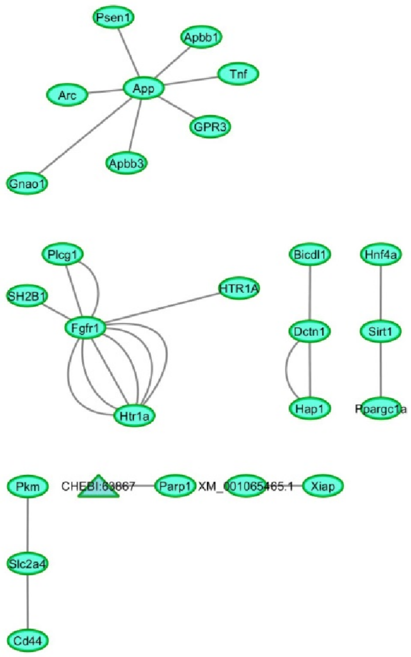

Supplemental Figure 2. IntAct graph resulting when NH synergic MoA-related proteins were used as seeds.

SUPPLEMENTARY TABLES AND HEADINGS

Table Supplementary 1. List of proteins in the PPI map corresponding to Endogenous Neuroprotection (see sup. dataset)

Table Supplementary 2. List of proteins in the PPI map corresponding to Neurodegeneration (see sup. dataset)
